# Prevalence and context of peer bullying among adolescents in schools from Shaqra City: public-schools surveillance in Shaqra, Saudi Arabia

**DOI:** 10.25122/jml-2024-0020

**Published:** 2024-07

**Authors:** Muath Alammar, Jalal Ali Bilal, Yasser Salem Saleh, Abdullah Mohammed Bin Hussain, Saif Munif Alshammari, Ishag Adam

**Affiliations:** 1 Department of Family Medicine, College of Medicine, Shaqra University, Shaqra, Saudi Arabia; 2 Department of Pediatrics, College of Medicine, Shaqra University, Shaqra, Saudi Arabia; 3 Department of Dermatology, College of Medicine, Shaqra University; 4 Department of Dermatology, Suez Canal University, Ismailia, Egypt; 5 College of Medicine, Shaqra University, Shaqra, Saudi Arabia; 6 Department of Obstetrics and Gynecology, College of Medicine, Qassim University, Buraidah, Saudi Arabia

**Keywords:** prevalence, age, bullying, schoolchildren, Shaqra, Saudi Arabia

## Abstract

Bullying in schools is a serious global health issue that jeopardizes youth and future adult health and negatively impacts academic outcomes. This cross-sectional study investigated the prevalence, forms, and associated factors of bullying among students aged 7-17 in public schools in Shaqra City, Saudi Arabia, where no prior data existed. A total of 372 students participated, with a median age of 11 years (interquartile range 9–14), of whom 187 were girls. 27 students (12.6%) reported being bullied in the past month, primarily through verbal abuse (89.4%), followed by physical bullying (10.6%). Cyberbullying was prevalent, with all students bullied experiencing it through social media, and some through texting (6 students, 1.6%) or email (3 students, 0.8%). Factors like student and parent age, student sex, school level, class level, nationality, chronic diseases, polygamy, and smartphone ownership were not associated with bullying. Cyberbullying and bullying affected 12.6% of students. Verbal bullying, the most common, occurred in restrooms and halls. Victims were hesitant to discuss their worries with anyone. Bullying incidents both within and outside of schools were more likely to result in positive adult intervention. Bullying among adolescents was attributed to factors such as physical strength and size. The possible reactions of bystander peers to a bullying incident include alerting the school administration and directly intervening independently.

## INTRODUCTION

School bullying is a significant global health concern with detrimental effects on youth well-being and academic achievement. Although bullying is a common occurrence in human interaction and was largely overlooked in the literature until recently, it is defined by three key factors: repetition, intentional harm, and an unbalanced power dynamic that favors the bully [1]. The Centers for Disease Control and Prevention (CDC) defines bullying as “any unwanted aggressive behavior(s) by another youth or group of youths who are not siblings or current dating partners, involving an observed or perceived power imbalance, and repeated or highly likely to be repeated multiple times. Bullying has the potential to cause physical, psychological, social, or educational harm to the targeted youth" [2].

Violence exposure, particularly during childhood or adolescence, can be traumatic and affects one’s ability to manage emotions and respond to stress throughout life [3]. Additional research is needed because bullying has extensive educational and health consequences during childhood and adolescence, as well as long-term effects in adulthood [4].

Bullying is manifested in different forms, such as physical, verbal, social, sexual, cyberbullying, and prejudicial, which targets individuals based on personal characteristics like race or religion [5]. The multitude of factors that influence bullying among adolescents are reported by a large-scale study from Ethiopia and well-characterized by the CDC. They can generally be summarized as age, gender, current substance use, emotional abuse, physical abuse, psychological distress, medical illness, social dynamics, school environment, and socioeconomic status. Subthemes such as dislike of school, racism, aggressiveness, and social isolation reflect the potential impact of bullying among Saudi adolescents [5–7].

Saudi Arabia has implemented several programs to combat bullying and violence, including the National Family Safety Program and, recently, the Rifq Program. Proposed initiatives also aim to address cyberbullying among high school students in Riyadh. These programs consider the psychological, social, and community-related aspects of this challenge [8].

The pooled prevalence of bullying victimization globally is reported at 30.5%, according to data from the World Health Organization (WHO) and the Global School-based Student Health Survey (GSHS). The prevalence in the Eastern Mediterranean Region was 45.1%, while Europe had the lowest (8.4%) [9]. In Saudi Arabia, reports indicate that the prevalence of bullying varies by region and data collection methods, ranging from 6.5% in Jeddah to 35% in the Jazan region [7,10–12].

Estimates of the prevalence of bullying among youth vary widely due to differences in assessment and definition. This variation makes it challenging to compare results across studies. Collecting data on bullying victimization is essential to continually monitor its prevalence, extent, and characteristics. This consistent data collection will facilitate research, prevention, and intervention efforts aimed at combatting bullying. Future studies should adopt the CDC’s standardized definition of bullying to enable easier comparisons with global studies using the same criteria [5].

The significance of this study is underscored by the increased use of technology, including the internet and mobile devices, which has created a new platform for bullying. No research has been conducted in Shaqra City to assess the prevalence of bullying among young people in the schools in the city. Such information could assist policymakers in deciding how to implement, evaluate, and manage public health practices.

This study aimed to determine the prevalence of bullying among students enrolled in Shaqra City public schools, the forms of bullying they experienced, and identify the risk factors.

## MATERIAL AND METHODS

### Study design, location, and population

This cross-sectional study was conducted from June 1 to July 31, 2023, among adolescents aged 7-17 years enrolled in public schools in Shaqra City, located in the Central region of Saudi Arabia. Shaqra province covers an area of 4110 km^2^ area and 27,848 residents. At the time of the study, there were twelve public schools in Shaqra City with four schools (two for girls and two for boys) at each grade level (primary, intermediate, and secondary).

### Sampling technique and sample size

A stratified random sampling method was used to select schools, classes, and students within each class. The sample size was determined using the OpenEpi Version 3 online software. The Shaqra Education Department recorded 2658 students registered in 12 schools at the time of the survey. Non-overlapping strata were used to stratify the schools. A confidence interval limit of 5% and a design effect of 1 were used to determine the hypothesized percent frequency of outcome component in the population (p), set at 35.0% [12] +/- 5 %. The sample size was calculated to be 370 at the 97% confidence interval. Two schools – one for girls and one for boys – were randomly chosen from all levels (primary, intermediate, and secondary).

### Data collection tool

The respondents and their families were given a definition of bullying based on the CDC definition, which included power imbalance, the intent to harm, and the distinction of the behavior from teasing [2]. After that, a structured questionnaire that was tested was used for the interview. The suggested questionnaire was modified from the one created by a collaborative effort between the United States Department of Education in Washington, D.C., and the Centers for Disease Control and Prevention in Atlanta, Georgia [2]. The questionnaire was translated into Arabic and subsequently used to collect responses. Prior to data collection, parents and teachers were informed about the study’s objectives, procedures, and participant rights, including voluntary participation and the ability to withdraw at any time without consequences.

In the schools for boys, intern doctors questioned the boys, and female teachers did the same in the schools for girls. A different medical intern interviewed both parents and their young students. The questionnaire included demographic data (core data) on age, sex, nationality, current grade level, ethnicity, and disability status. The questionnaire included extended data elements such as whether they were bullied in the preceding year, the context and type of bullying, the time and location of bullying, and the frequency and perceived reasons for bullying. Sexual behaviors and possible sexually transmitted diseases were excluded because they were deemed culturally inappropriate.

### Data handling and statistics

The data were handled confidentially, coded, and entered into SPSS version 21. Depending on the distribution, numerical data were expressed as mean (SD) or median (interquartile range). Nominal data are expressed as frequencies (%), and associations were tested using the chi-square test. Univariate analysis was performed to detect the relationship between the independent variables, namely, student and parent’s age, student’s sex, school level, class level, nationality, chronic diseases, polygamy, violence at home, and possession of a smartphone, and the dependent variable 'bullying status’ (bullied vs. not bullied). A logistic regression with backward elimination was performed using all independent variables. The adjusted ORs (AORs) and a 95% CI were calculated, and *P* < 0.05 was considered to indicate statistical significance.

## RESULTS

### Schools and participants

**Figure 1:**
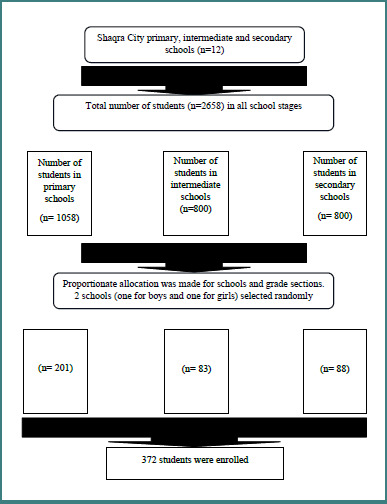
Flowchart of the enrolled schoolchildren of Shaqra city

At the time of the study, Shaqra City had 12 schools at each of the three levels (primary, intermediate, and secondary). There were 372 total participants recruited from six schools. Participants were chosen by their grade level and class level, as shown in Figure 1.

### Demographic characteristics

The median (interquartile IQ) age of the students (*n* = 372) was 11 (9–14) years, including 187 girls. There was no significant difference in age or nationality between boys and girls (*P* = 0.145 and 0.215, respectively). Only six (1.6%) students were non-Saudi, two were Egyptians, three were Syrians, and one was Yemeni. The fathers’ median age was 50 years (48–57), and the mothers’ median age was 45 years (40–50) years; however, neither was associated with bullying (*P* = 0.734 and 0.715, respectively).

### Prevalence of bullying

The prevalence of reported bullying of any type in this cohort was 12.6% (95% CI, 9.4% to 16.4%). All 47 victims experienced cyberbullying. More boys reported being bullied than girls (29 boys [7.^8^%] vs. 18 girls [4.^8^%]), although the difference was not significant (*P* = 0.087). Bullying was recorded only among Saudi students in primary and secondary schools, specifically within the first and second years. These students had parents whose education was at the secondary school level or below. The most common occupation among the fathers of schoolchildren who experienced bullying was teaching, whereas homemaking was the most common occupation among their mothers. Students from non-polygamous families reported bullying. However, none of the aforementioned characteristics were significantly more common among children who reported bullying than among children who did not (Table 1).

**Table 1:** Demographic characteristics and bullying among schoolchildren in Shaqra (*n* = 372)

Variable		Frequency (%)			*P* value
		Bullied	Not bullied	Total	
**Sex**	Boys	29 (15.7)	156 (84.3)	185 (49.7)	0.087
	Girls	18 (9.6)	169 (90.4)	187 (50.3)	
**Nationality**	Saudi	47 (12.8)	319 (87.2)	366 (98.4)	0.201
	Non-Saudi	0 (0.0)	6 (100)	6 (1.6)	
**Father education level**					
	Secondary school	26 (12.9)	175 (87.1)	201 (54)	0.160
	University	7 (8.4)	76 (91.6)	83 (22.3)	
	Higher education	14 (15.9)	74 (84.1)	88 (23.7)	
**Mother education level**					
	Secondary school	20 (14.5)	120 (86.5)	138 (37.2)	0.681
	University	24 (12.4)	170 (87.6)	194 (52.4)	
	Higher education	3 (7.9)	35 (92.1)	38 (10.4)	
**Father occupation**					
	Retired	14 (13.7)	88 (86.3)	102 (27.4)	0.356
	Other	6 (7.5)	74 (92.5)	80 (21.5)	
	Manager	7 (9.3)	68 (90.7)	75 (20.2)	
	Policeman	12 (17.1)	58 (82.9)	70 (18.8)	
	Teacher	8 (17.8)	37 (82.2)	45 (12.1)	
**Mother occupation**					
	Housewife	23 (14.3)	138 (85.7)	161 (43.3)	0.964
	Teacher	17 (12.5)	119 (87.5)	136 (36.6)	
	Retired	3 (9.7)	28 (90.3)	31 (8.3)	
	Manager	3 (10.7)	25 (89.3)	28 (7.5)	
	Other	1 (6.3)	15 (93.7)	16 (4.3)	
**Polygamy**					
	Yes	5 (11.9)	37 (88.1)	42 (11.3)	0.880
	No	42 (12.7)	288 (87.3)	330 (88.7)	
**School level**					
	Primary	26 (12.9)	175 (87.1)	201 (54)	0.333
	Intermediate	7 (8.4)	76 (91.6)	83 (22.3)	
	Secondary	14 (15.9)	74 (84.1)	88 (23.7)	
**Class level**					
	First (all grades)	15 (13.2)	99 (86.8)	114 (30.6)	0.172
	Second (all grades)	11 (10.1)	98 (89.9)	109 (29.3)	
	Third (all grades)	7 (10.3)	61 (89.7)	68 (18.3)	
	Fourth (primary school)	4 (10.0)	36 (90%)	40 (10.8)	
	Fifth (primary school)	10 (24.4)	31 (75.6)	41 (11.0)	

### Type and context of bullying

Of the 47 victims, 42 (89.4%) reported verbal bullying (calling inappropriate names or phrases), whereas the remaining (10.6%) reported physical intimidation (pushing, slapping, punching). All of them were bullied electronically. Almost all respondents (97.6%) reported that cyberbullying occurred through social media, six (1.6%) through direct texting, and only three (0.8%) through emailing. However, only 47 (12.6%) were victims of cyberbullying.

Of the 47 victims of bullying, the majority (43, 91%) stated that the frequency of bullying was once or twice per month, mostly involving 1-3 peers. Almost all bullying events (95.4%) occurred within the school vicinity, mostly in corridors (54.8%) and restrooms (17.5%) and with decreased frequency during sports and in the cafeteria. The majority of schoolchildren (*n* = 299, 80.4%) were not fearful of being bullied. Thirty-two (8.6%) respondents stated that they would not talk to anyone about being bullied because they were afraid of being judged. However, few respondents stated that they had expressed their concerns to their parents, teachers, friends, and siblings (Table 2).

**Table 2:** Characteristics and context of bullying among schoolchildren in Shaqra schools

Variable		Frequency	%
**How frequently have you been bullied?**			
	Once or twice/month	31	66.0
	Regular (1-2/week)	12	25.5
	Everyday	4	8.5
	Total	47	100.0
**Type of bullying**			
	Calling names/inappropriate phrases	42	89.4
	Physical	5	10.6
	Total	47	100.0
**Number of peers who bullied you during one occasion**			
	1-2	27	57.4
	2-3	12	25.5
	3-4	2	4.3
	>4	6	12.8
	Total	47	100
**Where bullying occurs to you or to others**			
	Corridors	204	54.8
	Restrooms	65	17.5
	Electronic	47	12.6
	During sport	24	6.5
	In the bus	17	4.6
	Cafeteria	15	4.0
	Total	372	100.0
**Absence due to bullying in this academic year**			
	Never	344	92.5
	Once or twice/week	13	3.5
	Once or twice/month	3	0.8
	Once or twice/3 months	3	0.8
	Once or twice/6 months	6	1.6
	Everyday	3	0.8
	Total	372	100.0
**Do you fear of being bullied**			
	Yes	73	19.6
	No	299	80.4
	Total	372	100.0
**Have you talked to anyone about being bullied?**			
	I have not been bullied	319	85.8
	No, I am scared I will be judged	32	8.6
	Yes, I have expressed my concerns to my parents	11	3.0
	Yes, I have expressed my concerns to my teachers	4	1.1
	Yes, I have expressed my concerns to my friends	4	1.1
	Yes, I have expressed my concerns to my siblings	2	0.5

### Family violence, bullying others, and perceived reasons for bullying

**Figure 2:**
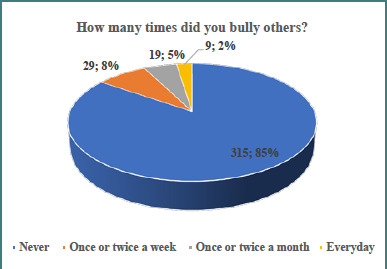
Frequency of bullying behavior among students in Shaqra schools

Only five (1.3%) students reported family violence. In response to occasions of bullying others, the majority (84.7%) of the schoolchildren reported that they had never bullied anyone before, and the remaining reported once or twice per week or month and every day, as depicted in Figure 2. Among all respondents (*n* = 372), 37 (9.9%) were bullies and not victims whereas 20 (5.4%) experienced bullying, i.e., they bullied others as well as were victims of bullying (*P* <0.001). There was no significant sex difference among them (*P* = 0.177).

**Figure 3:**
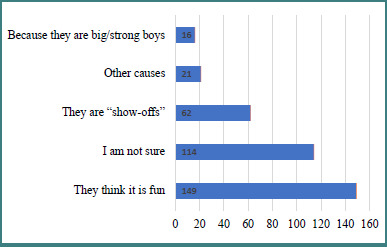
Students’ perceptions on the reasons behind bullying

When asked about the motivation of bullies, more than 40% of the students considered that bullies were doing it for the sake of fun while more than a third believed that they were not sure. Other opinions were also expressed, as Figure 3 illustrates.

### Students’ perception of responses of adult witnesses and peers’ bystanders to bullying

The majority of adults who witnessed the incident outside (86.6%) and within the school (59.7%) reported taking positive action, intervening to stop the aggressiveness, administering direct punishment (20.4%), or reporting it (9.4 %). Peer bystanders responded that they would report to school authorities (41.7%) and act on their own (32.3%), and almost more than a fifth (22.8%) responded that they did nothing in response to witnessing a bullying event. Seventy-eight percent of peers who were bystanders were reported to positively intervene in the event (Table 3).

**Table 3:** Perspectives of children on how witnesses in Shaqra schools responded to bullying incidents

Variable		Frequency	%
**What was the response of adults anywhere to bullying**			
	Intervening	322	86.6
	Watching	50	13.4
**What do adults in your institution do when they see bullying?**			
	Nothing, they turn a blind eye	150	40.3
	Stop it immediately	107	28.8
	Punish them harshly	76	20.4
	Report to school authorities	35	9.4
	Call the police	4	1.1
**If you saw someone being bullied at school, would you**			
	Report to school authorities	155	41.7
	Take matters in your own hand	120	32.3
	Do nothing	85	22.8
	Tell your parents	2	0.5
	Call the police	1	0.3
	Other	9	2.4
**Response of peers to bullying**			
	Intervening	290	78
	Watching	82	22

### Factors associated with bullying

According to our univariate analysis, students’ and parents’ age, sex, school level, class level, nationality, chronic diseases, polygamy, and possession of a smartphone were not associated with bullying. Violence at home was associated with bullying (OR=11.01; 95% CI, 1.79-67.7; *P* = 0.010). However, multivariate analysis revealed that none of these factors was associated with bullying (Table 4).

**Table 4:** Univariate and multivariate analysis of factors associated with peer bullying among schoolchildren in Shaqra (n = 372)

Variable	Univariate			Multivariate		
	OR^*^	95% CI^**^	*P* value	AOR^***^	95% CI	*P* value
**Student’s age**	1.06	0.96-1.17	0.222	1.29	0.98-1.70	0.069
**Father age**	0.96	90.0-1.02	0.260	0.96	0.89-1.03	0.251
**Mother age**	1.01	93.0-1.08	0.864	0.99	0.93-1.08	0.998
**Sex**	1.75	93.0-3.27	0.082	1.60	0.82-3.15	0.171
**School level (primary vs secondary)**	0.79	0.39-1.60	0.785	0.23	0.03-1.89	0.176
**Class level**	1.10	0.57-1.96	0.850	1.11	0.25-5.07	0.889
**Nationality**	1.64	0.84-0.91	0.442	180	0.0-‒179	0.999
**Chronic diseases**	2.23	0.85-5.88	0.104	1.58	0.53-4.66	0.411
**Polygamy**	0.93	0.35-2.49	0.880	1.24	0.44-3.49	0.681
**Violence at home**	11.01	1.79-67.7	0.010	6.92	0.96-49.99	0.055
**Had smartphone**	1.12	0.32-3.86	0.862	0.78	0.21-2.92	0.715

* OR, odd ratio; **CI, confidence interval limit, ***AOR, adjusted odd ratio.

## DISCUSSION

This study aimed to estimate the rate of peer bullying in schools within a small city characterized as a mixed urban-rural community in Saudi Arabia. According to the current study, 12.6% of respondents were victims of both traditional and cyberbullying in the preceding year. However, UNESCO reported a much higher global prevalence (32%) of bullying in the previous month among schoolchildren. The prevalence of bullying varies significantly across the world, with 22.8% of children being victimized in Central America, 25.0 % and 31.7 % in Europe and North America, respectively, and 48.2% in Sub-Saharan Africa [4]. Biswas *et al*.[8], based on GSHS data, reported a pooled prevalence of bullying victimization of 30.5%; the highest was observed in the Eastern Mediterranean region (45.1%), and the lowest was in Europe (8.4%) [9]. The school systems of 83 countries, including Saudi Arabia, took part in the Trends in International Mathematics and Science Study (TIMSS). According to TIMSS, approximately 43% of schoolchildren worldwide experience bullying at least monthly, and more than half of Saudi participants are bullied at least monthly [13]. The prevalence of bullying victimization varies widely across schools in Saudi Arabia, with values as high as 89.2% and as low as 6.5% [10,12,14–17]. Because different possible criteria were used to describe bullying, the prevalence in this study should be interpreted with caution when compared to international and local reports. The variation in bullying prevalence has been noted even within schools in the same country or region [13]. Furthermore, definitions of bullying-like phenomena vary linguistically and may be influenced by what is deemed legitimate from a cultural standpoint [18]. This study’s relatively low prevalence of bullying compared to national and international figures may be because almost half of the participants were from primary schools where the age range was lower (median 9-14) than in the other studies. It is known that adolescents sometimes do not report bullying incidents because they fear threats or embarrassment and may prefer to tell friends rather than teachers or parents [8]. This may result in underreporting of bullying. Additionally, when parents answer questionnaires on behalf of their children, they may not always provide accurate responses, which could further contribute to the lower prevalence observed [15]. Social and cultural factors may also influence the national prevalence of bullying [9].

In this study, boys were bullied more than girls, but the difference was not significant. Gender differences have been observed in international and Saudi studies across regions, countries, and school levels [7,9,10,12].

Verbal bullying was the most commonly reported type of bullying among victims (89.4%) followed by physical. Similar studies from Saudi Arabia reported that verbal abuse was the most common type of bullying. However, the rates of verbal abuse differed. Elmahdy *et al*. [12] reported a lower rate (75.6%) of verbal bullying compared to this study, and Alsaleem *et al*. [19] reported an even lower rate than both studies. The CDC defines verbal bullying as harmful oral or written communication, taunting, calling names, making inappropriate sexual comments, or threatening another youth [2]. Different definitions and, hence, reports of verbal bullying among different settings might explain the variation in the prevalence of verbal bullying among different studies. The most commonly reported forms of verbal bullying among Saudi students were calling names and verbal insults [7].

The cyberbullying victimization rate (12.6%) was similar to traditional bullying in this study. However, most of the respondents (97.6%) stated that cyberbullying did occur either through social media, direct texting, or email. The 47 students who reported traditional bullying also reported cyberbullying. Arasheed *et al*. [20], reported an 18% rate of cyberbullying among high school students though their study involved an older population (15-19 years). Given the prevalence of technology use among Saudi adolescents, cyberbullying is unavoidable. Rumor spreading, social isolation, and blackmailing have all been reported to have occurred in cyberspace [7]. There is still uncertainty about what construct is being assessed by current cyberbullying measures. A clear definition of cyberbullying is critical for establishing measurement validity. This has hampered the reliability of studies on the prevalence, incidence, outcomes, and thus, interventions associated with cyberbullying, as well as the ability to make meaningful comparisons with traditional bullying [21].

The most common forms of bullying in this cohort were cyberbullying, verbal bullying, and physical bullying. Cyberbullying was reported to occur through social media, direct texting, and email. Since adolescents in Saudi Arabia are frequent users of technology, cyberbullying often takes place in the form of spreading rumors, social isolation, and blackmailing. Verbal bullying was observed in the form of name-calling and using inappropriate language. Instances of verbal insults, such as calling someone “fat” or not speaking to each other politely, were reported. The low reporting of physical bullying in this cohort may be due to adults underestimating it and considering it as rough play [7]. Several important factors have been shown to contribute to bullying behaviors in Saudi Arabian teenagers. Firstly, schools lack a safe and secure atmosphere in schools, which fails to provide the necessary protection against bullying. Secondly, the lack of sports and extracurricular activities in schools exacerbates the problem. Thirdly, the inconsistent approach taken by school personnel in dealing with student behavior is a contributing factor. Fourthly, the unhealthy relationship between students and their teachers is a significant issue. Finally, insufficient communication and blame-shifting between families and schools are also factors that contribute to bullying behaviors in Saudi Arabian teenagers [7]. A subsequent study established a relationship between seeking attention, anger, and bullying [22]. Research has shown that younger children who have a negative attitude towards school or those who frequently skip school are at a higher risk of being bullied. Additionally, students who receive poor grades as a result of bullying, or those who are targeted due to their good academic performance and interest in school, are also more likely to become victims of bullying. The parents’ report on bullying also highlights that children with physical features such as dentofacial appearance are more prone to being targeted by bullies [15]. Furthermore, bullying victimization among Ethiopian adolescents was substantially correlated with male sex, current substance use, emotional and physical abuse, psychological distress, and having a medical condition [6].

The majority of bullying incidents (95.4%) occurred in school restrooms and hallways. When asked to name locations where bullying frequently occurs, respondents reported similar contexts but in varying proportions. Even if bullying takes place off school premises, there is often spillover into the school environment [23].

Only 5% of bullying victims expressed concerns about being bullied by anyone. In a longitudinal sample in a different setting, almost all (55.4%) participants had told several people about being victimized at school [24]. Although the mean age was similar to that in Blomqvist *et al*.’s, cohort, social cultures, and norms differed. Almost 9% of the respondents stated that they might be judged if they expressed their concerns about being bullied.

In this study, more than 9% of respondents were found to be bullies, compared to just 5% who were bully-victims. Moreover, there was no significant difference between boys and girls. A similar rate was reported in Jazan, southern Saudia Arabia, though this was a response from bullies and bully-victim [12]. Potard *et al*. [25] reported slightly different data, noting significant sex differences concerning bullying roles. They found that pure victims were more likely to be girls, whereas bully-victims were more likely to be boys. Potard *et al*. suggested that boys are more likely than girls to experience bullying and reported similar results to ours, except that they found boys were more often bullies than girls. This difference may be attributed to the fact that boys tend to exhibit more aggressive behavior than girls.

Furthermore, slightly fewer than half of the students thought the most likely reason for bullying was ‘simply for fun’. A relationship between bullying perpetuation and social goals (status/power) as a goal-oriented method to acquire status and power was reported by a recent meta-analysis [26].

The majority of teachers and other adult staff in this cohort, as well as most people outside the institutions, were thought to have positive attitudes response to bullying. Adults who observe bullying frequently have assumptions about its traits and frequency. Some adults consider bullying to be a common rite of passage for children rather than a bullying action [27]. In an interview with youth, adults responded to bullying in three ways, which occasionally interacted and overlapped. This could take the form of a verbal or violent retort or involve doing nothing and avoiding or ignoring the bullying [27]. The majority of bystanders in this survey (41.7%) said they would notify the school administration, while a third would take action on their own, and more than a fifth would not intervene. In a similar setting, positive actions were reported among 33% of the bystanders [10]. Young people who observe bullying can significantly contribute to its continuation or disruption. Because witnessing bullying has harmful impacts on children, it is crucial to monitor the percentage of children who experience bullying. Additionally, monitoring the proportion of children and adolescents who often witness bullying aids in determining the severity of the issue [2].

Violence at home was the only associated factor for being a victim of bullying; however, when adjusting for all other factors, none of the factors was associated with victimization. This study did not examine the whole range of variables that could positively or negatively impact bullying. A considerable risk of cyberbullying was shown to be associated with male sex, poorer mental health, being bullied, and smoking [14]. Boys were more likely than girls to experience victimization, and aggressive behaviors were much more prevalent at the middle stage than at the superior stage [28].

We acknowledge several limitations in our study. First, the use of cross-sectional data rather than longitudinal data is a significant limitation. Second, the conclusions of this study may not apply to all of Saudi Arabia because they were based on the limited population of a small, localized city. Students from larger populations might have different concerns and opinions. A long-term longitudinal study with direct observation and stringent standards without knowledge of the students in schools could produce better results. Third, we were unable to report on sexual bullying due to the social stigma associated with discussing its nature. Finally, additional demographic, personal, and psychological factors could have been examined. Including these factors might have provided a more comprehensive understanding of the associations related to bullying.

Bullying is a significant issue that requires the establishment of prevention policies and procedures in Saudi schools. To target cyberbullying, professional intervention using group counseling can be implemented. Schools should have clear, evidence-based rules against bullying, especially verbal bullying. Interventional training programs to improve student supervision, particularly outside classrooms in corridors and cafeterias, should be developed. Existing programs like the Saudi National Family Safety Program and the Rifq Program can be updated to emphasize the role of family violence in bullying and to target factors contributing to bullying, in collaboration with the Ministry of Education.

## CONCLUSION

Cyberbullying and bullying affected 12.6% of adolescents in Shaqra schools. Verbal bullying occurred in restrooms and halls. Victims were hesitant to discuss their worries with anyone. Being a bully was less common than being a victim, and bullying often occurred for amusement. Other contributing factors included being a big/strong boy, being a show-off, and possible violence at home. Bullying incidents both within and outside of schools were more likely to result in positive adult intervention. The likely reactions of peers’ bystanders to a bullying incident include alerting the school administration and directly acting on their own. Establishing rules and policies in school culture, in addition to national-level programs, can lead to a decrease in bullying.

## Personal thanks

We appreciate the students’ and their families’ involvement in this study. We are grateful for the support provided by the Shaqra Department of Education and the Deanship of Scientific Research at Shaqra University. We are deeply thankful to the female teachers who helped collect data from girl schools. Without the efforts of the instructors, especially in the girls’ schools, this study would not have been possible.
